# Long term follow-up report of cardiac toxicity in patients with multiple myeloma treated with tandem autologous hematopoietic stem cell transplantation

**DOI:** 10.2478/raon-2013-0019

**Published:** 2013-05-21

**Authors:** Mirta Kozelj, Samo Zver, Vesna Zadnik

**Affiliations:** 1 Department of Cardiology, University Medical Centre Ljubljana, Ljubljana, Slovenia; 2 Department of Haematology, University Medical Centre Ljubljana, Ljubljana, Slovenia; 3 Institute of Oncology Ljubljana, Ljubljana, Slovenia

**Keywords:** cardiotoxicity, cyclophosphamide, melphalan, echocardiography

## Abstract

**Background:**

Tandem autologous hematopoietic stem cell transplantation (ta-HSCT) is a standard treatment for multiple myeloma (MM). Patients receive a high-dose cyclophosphamide (CY), followed by two myeloablative cycles of melphalan (MEL). There are scarce data about long term cardiotoxicity.

**Patients and methods.:**

We studied 12 patients (62.25 ± 8.55 years) six years after the completion of MM treatment with ta-HCST. Late cardiotoxic effects were evaluated clinically and echocardiographically.

**Results:**

None of the patients developed clinical signs of heart failure, all were in sinus rhythm and NT-pro BNP concentration was elevated (778 ± 902.76 pg/mL). The left ventricular (LV) size remained normal. The LV ejection fraction did not decrease (73.75 ± 5.67%, 69.27 ± 6.13%, p = NS). The LV diastolic function parameters (E, A, ratio E/A and A/a) did not change significantly. In tissue Doppler parameters we observed a nonsignificant decrease in E_m_ (10.26 ± 2.63 cm/s, 7.57 ± 1.43 cm/s) and S_m_ velocities (8.7 ± 0.87 cm/s, 7.14 ± 1.17 cm/s, p = NS). The E/E_m_ values were in an abnormal range (8.66 ± 1.05, 10.55 ± 2.03).

**Conclusions:**

The treatment of MM with ta-HSCT, during which patients receive a high dose CY followed by two myeloablative cycles of MEL, causes mild, chronic, partially reversible and clinically silent cardiotoxic side-effects. However, ta-HSCT in patients with MM is a safe regarding cardiotoxic side effects, but, because of increasing life expectancy needs long term attention.

## Introduction

Autologous hematopoietic stem cell transplantation (HSCT) is one of the most efficient therapies for patients with multiple myeloma (MM).^1^ The intensification of the treatment with double autologous HSCT very likely results in increased survival rates for MM patients.^2^,^3^ In the era of novel agents, upfront tandem autologous hematopoietic stem cell transplantation (ta-HSCT) may not be as strong a treatment modality as some years ago.^4^ However, the procedure remains a powerful tool against the disease and if not performed upfront, it represents a reliable treatment option at the time of disease relapses.

Because our study started in the era before novel agents were available, included patients received vincristine, epirubicin, dexamethasone (VED) induction chemotherapy, followed by cyclophosphamide with granulocyte colonies stimulating factor (G-CSF) as part of the stem cell mobilization and collection regimen and first myeloablative cycle of melphalan (200 mg/m^2^). Patients who did not respond completely underwent a second ta-HSCT with the same dosage of melphalan within the next few months. They, thus, received a high dose cyclophosphamide and two myeloablative dosages of melphalan during a short period of time. Both chemotherapeutic agents may exert cardiotoxic side effects.^5^ Namely, neurohormonal activation of heart failure was observed during the treatment. Doppler echocardiography studies revealed the worsening of the left ventricular diastolic function and the occurrence of functional mitral regurgitation. We have found no reports on chronic cardiotoxic effects of cyclophosphamide and melphalan administered as a part of the treatment with ta-HSCT.

Cyclophosphamide doses greater than 1.5 g/m^2^ may be associated with the acute cardiotoxic risk.^6^ Cardiac toxicity is manifested as acute-onset heart failure and/or myocarditis occurring within 1 to 10 days after the administration of the first dose of cyclophosphamide. Echocardiography revealed reversible reduction in the left ventricular ejection fraction.^7^

Cardiac toxicity of melphalan may manifests itself as paroxysmal artial fibrillation.^8^ The use of melphalan in combination with purine analogue fludarabine has been reported to produce acute-onset left heart failure, even in individuals with no history of prior heart disease.^9^ Prospective studies conducted to date have not confirmed long-term cardiotoxic effects of melphalan, yet cardiotoxic side effects of the drug cannot be ruled out completely.

In the former echocardiographic study of acute, subacute and early chronic cardiotoxic side effects of cyclophospamide and melphalan in the treatment MM with ta-HSCT, we confirmed a diastolic left ventricular dysfunction and the increase incidence of mitral regurgitation.^5^

Therefore, this study was undertaken to evaluate possible late cardiotoxic effects of the aforementioned drugs and prospectively investigate the cardiac function and indicators of heart failure in individuals six years after the treatment with ta-HSCT.

## Patients and Methods

### Patients

We studied 12 surviving patients (out of 30 patients initially treated) six years after successful autologous HSCT because of MM: 7 men and 5 women, aged 53 to 75 (62.25 ± 8.55). None of them had at presentation, nor later on in the course of the disease, the extramedullary form of MM involving the chest, cervical or thoracic spine that would require the treatment by irradiation of the thoracic cavity.

### Study design

During the year 2005, thirty patients with MM underwent ta-HSCT. The trial started four weeks after the completion of the last out of three cycles of VAD chemotherapy, *i.e.* prior to the administration of cyclophosphamide at a dose of 4 g/m^2^ as a part of the stem cell mobilization/collection regimen. Prior to the initiation of cyclophosphamide therapy, physical examination and baseline echocardiography (phase 0) were carried out. Two months after the cyclophosphamide therapy, the administration of the first myeloablative cycle of melphalan (200 mg/m^2^) was administered. Four months after the administration of the first cycle of melphalan, the patients received the second myeloablative cycle of melphalan (200 mg/m^2^) as a part of the ta-HSCT. Three months after the patients had received the second cycle of melphalan, we made the final assessment of cardiac toxicity of cyclophosphamide and two successive myeloablative dosages of melphalan, based on the last physical examination and echocardiography (phase 1). In the year 2007, two years after the completion of autologous HSCT, the echocardiographic study was repeated (phase 2) and in the year 2011, six years after double autologous HSCT, we finished the trial with the fourth echocardiographic follow up (phase 3). At each step of the study we examined the patients regarding clinical signs and symptoms of heart failure and performed ECG. N-terminal prohormone of brain natriuretic peptide (NT-proBNP) serum concentrations were measured at the very end of the study.

### Echocardiography

Each patient was evaluated at each phase with two-dimensional, conventional Doppler and tissue Doppler echocardiographic studies. All examinations at all times were carried out by the same examiner. We determined left ventricular end-diastolic (LV EDD), left ventricular systolic diameter (LV ESD), calculated the left ventricular ejection fraction (LV EF), and assessed the left atrial transversal and longitudinal diameters. The systolic function of the left ventricle was assessed also by the velocity time integral of the left outflow tract (VTI). The left ventricular diastolic function was assessed by measuring diastolic mitral inflow velocities (E and A-wave), pulmonary vein systolic (s-wave), diastolic (d-wave) and atrial reversal (a-wave) velocities. The E/A velocities index and A/a duration ratio were calculated as markers of the diastolic left ventricular function. In addition, tissue Doppler echocardiography of the mitral annulus was used for assessing the left ventricular systolic and diastolic function. Mitral annulus systolic (S_m_) and early mitral annulus diastolic tissue velocity (E_m_) were measured. The E/E_m_ index was calculated as a marker of increased left atrial pressure. We used the mean value of three consecutive measurements for each of the parameters examined. Each examination included the evaluation for potential mitral regurgitation and pericardial effusion.

### Statistical analysis

All analysed variables are distributed normally and are presented as the mean ± standard deviation. The associations between NT-proBNP serum concentration and individual echocardiographic parameters were evaluated by calculating Pearson correlation coefficients. Repeated measures general linear model (GLM) with Bonferroni’s multiple comparisons test was used to assess the possible significance of changes in each echocardiographic parameter in successive time points. A p value of 0.05 or less was considered statistically significant. Statistical analyses were performed with the SPSS software package (version 13.0).

## Results

### Patient data

The study included 12 surviving patients with MM (7 men and 5 women) after successful double autologous HSCT. Eighteen out of 30 patients initially included in the study died because of MM progression during the period of six years. None of them complained of heart failure symptoms, nor did they manifest signs of it during the period mentioned.

All 12 enrolled surviving patients had normal baseline cardiovascular status. None of them developed clinically overt heart failure at any point in the study and electrocardiographic recordings disclosed no dynamic changes or rhythm abnormalities. In phase 3, the average value of NT-proBNP was 778 ± 902.76 pg/mL (range 124 – 2724 pg/mL).

### Echocardiography

The echocardiographic parameters are indicated in Table 1.^10^, ^11^ The left ventricular and atrial size did not increase significantly in any phase of the study and remained within normal limits. Standard parameters for the left ventricular systolic function and LVEF did not decrease. The VTI decreased significantly between phases 0 and 2 (p = 0.04) but it recovered as late as six years after the treatment with double autologous HSCT. However, the values were within normal. The S_m,_ a very sensitive tissue Doppler parameter, mildly decreased throughout the study and finally the values dropped under the lowest reference values.

The standard parameters identifying the diastolic left ventricular function by mitral flow velocities (E, A, E/A, A duration) did not change significantly during the six years of observation. Nevertheless, the E/A ratio reached the criteria of the abnormal relaxation in phases 1 and 2 and was restored in the phase 3. The duration of a wave in pulmonary vein flow decreased from phase 1 to 2 significantly (p = 0.04). The ratio A/a improved from phase 1 to 2 significantly (p = 0.001). No statistical differences regarding the baseline values could be found in other pulmonary vein parameters. In tissue Doppler parameters we observed a mild progressive decrease of E_m_ velocities without statistical significance. The E/E_m_ values were in an abnormal range for this age group from the phase 1 on. The E/E_m_ ratio showed the gradual increase during all phases with statistical significance (p=0.01) and was in an abnormal range from phase 1 on. The only positive correlation was found between serum NT-proBNP concentration and E/E_m_ (p = 0.009).

The frequency of mitral regurgitation and pericardial effusion did not reach statistically significant difference between the baseline study and phase 2 or 3.

There were no statistically significant differences between results of the baseline study (phase 0) and the results of the long term study in any of the observed parameters.

## Discussion

Ta-HSCT is an efficient and frequently used treatment modality regimen for patients with MM. Patients treated with ta-HSCT develop immediate but transient, mostly reversible and clinically non-overt neurohormonal activation of heart failure in each phase of their treatment. Early post-treatment abnormalities including diastolic dysfunction with abnormal left ventricular relaxation, elevated left atrial pressure and functional mitral regurgitation are detected echocardiographicaly.^12^ Very little is known about eventual chronic cardiac toxicity of this treatment regimen, which includes cyclophosphamide and a high dose melphalan as part of stem cell mobilisation and collection, followed by two autologous HSCT. There are no long term trials on cardiotoxicity in this treatment modality.

In this long term study, we followed up 12 survivors out of 30 treated MM patients with ta-HSCT. The rest, 18 out of 30 patients initially included, died of MM progression and did not suffer from symptoms of heart failure at any time. In the 12 currently alive MM patients, the clinical examination did not reveal any signs of heart failure.

ECG did not show any rhythm disturbances. They were all in sinus rhythm. The left atrial and ventricular size and left ventricular ejection fraction did not show any significant changes in a period of six years after the completion of therapy and all parameters were in the range of normal values. This result is as expected, because these are rather nonsensitive parameters of cardiotoxicity. The VTI, Doppler echocardiographic marker of systolic left ventricular function, was in the range of normal values as well, although it showed a mild decrease for the period of the follow-up but recovered to baseline values within six years. The tissue Doppler parameters are more sensitive and we noticed a gradual decrease in S_m;_ the values did not reach normal range even after the completion of the therapy. The S_m_ decrease was not statistically significant, probably because of the small number of patients. Therefore, we can conclude that there are some indices for a mild systolic dysfunction at long term follow-up. We suggest that stress echocardiography may be helpful in solving this question.

Diastolic dysfunction parameters are much more reliable and sensitive markers of cardiotoxicity than parameters of the left ventricular systolic function, as has been proven for acute cardiotoxicity in many Doppler echocardiographic studies.^12^–^14^ The E/A ratio reached the criteria of the abnormal relaxation after the treatment and we noticed its recovery to the normal ratio not earlier than at the six-year follow-up. The changes in duration of the transmitral A wave and the pulmonary a wave also showed a pattern significant for the elevated left atrial pressure, especially the A/a ratio. Despite that, the A/a ratio normalized as early as two years after the completion of the therapy. At the end of the follow-up there were no significant differences in Doppler echocardiographic diastolic function markers between the baseline study and the final study. So the conventional echocardiographic methods exclude late complications of cardiotoxic therapy with a high dose cyclophosphamide and melfalan in the context of ta-HSCT. In recent years, tissue Doppler echocardiography has been used with the aim to identify acute and chronic subclinical alterations of the left ventricular function in patients treated by antracyclines, while we have very little experience with tissue Doppler echocardiography in patients treated by other chemotherapeutic agents.^15^ In our group of patients, we noticed abnormal E_m_ velocities from the completion of therapy on and further on the values reached neither normal nor baseline levels at the end of the study. We believe that the decrease of E_m_ velocities did not reach statistical significance because of the small number of patients. The E/E_m_ ratio, a marker of elevated filling pressure of the left ventricle, was increasing throughout the follow-up and at the end of the study exceeded the value immediately after the completion of the therapy; it was in the abnormal range.^11^ This result is suggestive of the elevated filling pressures late after the therapy. Positive correlation in the serum concentration of NT-proBNP and E/E_m_ supports this fact. The highest values of NT-proBNP were present in two patients with the lowest E/E_m_.

## Conclusions

We conclude that the treatment of patients with MM with ta-HSCT, in which the patients receive a high dose cyclophosphamide and two myeloablative dosages of melphalan, causes mild, chronic, partially reversible, but clinically silent cardiotoxic side effects. The most reliable parameters were NT-proBNP and tissue Doppler parameters of diastolic function of the left ventricle. To determine this with certainty, we need studies with a greater number of patients and stress echocardiographic studies. Long term studies with a longer period of observational time would not improve our knowledge of long term side effects because of the influence of age on Doppler and especially on tissue Doppler parameters.

However, our study shows that ta-HSCT in patients with MM represents a safe treatment modality regarding cardiotoxic side effects, but because life expectancy in these patients is increasing, needs a long term follow up.

## Figures and Tables

**TABLE 1:** Echocardiographic characteristics

	**Phase 0**	**Phase 1**	**Phase 2**	**Phase 3**	**Signif phase 1:2 p**	**Signif phase 1:3 p**	**Signf phase 0:2 p**	**Signif phase 0:3 p**
**LV EDD (cm)**	4.69±0.4	4.45±0.63	4.63±0.38	4.65±0.85	NS	NS	NS	NS
**LV ESD (cm**)	2.65±0.3	2.7±0.35	2.6±0.31	2.87±0.56	NS	NS	NS	NS
**LA tr (cm)**	4.06±0.54	3.85±0.53	3.93±0.45	4.08±0.92	NS	NS	NS	NS
**LA long (cm)**	4.4±0.43	4.42±0.47	4.34±0.75	4.67±0.79	NS	NS	NS	NS
**LV EF (%)**	73.75±5.67	68.66±9.3	75±4.41	69.27±6.13	NS	NS	NS	NS
**VTI (cm)**	25.45±4.09	21.35±3	22.66±3.47	24.28±2.51	NS	0.05	0.04	NS
**E (m/s)**	0.86±0.16	0.76±0.16	0.74±0.18	0.79±0.13	NS	NS	0.02	NS
**A (m/s)**	0.88±0.16	0.89±0.16	0.82±0.21	0.82±0.2	0.05	NS	NS	NS
**E/A**	1.03±0.34	0.88±0.26	0.97±0.43	1.05±0.42	NS	NS	NS	NS
**A/a**	1.3±0.28	0.98±0.22	1.34±0.3	1.28±0.25	0.001	NS	NS	NS
**s (m/s)**	0.72±0.14	0.67±0.09	0.65±0.15	0.61±0.11	NS	NS	NS	NS
**d (m/s)**	0.55±0.16	0.48±0.13	0.47±0.12	0.51±0.18	NS	NS	NS	NS
**s/d**	1.38±0.45	1.5±0.45	1.44±0.43	1.34±0.55	NS	NS	NS	NS
**S**_m_**(cm/s)**	8.7±0.87	8.1±2.68	7.77±0.97	7.14±1.17	NS	NS	NS	NS
**E**_m_**(cm/s)**	10.26±2.63	7.78±1.44	7.68±1.58	7.57±1.43	NS	NS	NS	NS
**E/E**_m_	8.66±1.05	10.43±1.69	9.69±1.79	10.55±2.03	NS	0.01	NS	NS
**PE (N)**	1	2	2	3	NS	NS	NS	NS
**MR (N)**	4	6	1	3	NS	NS	NS	NS

LV EDD = left ventricular end diastolic volume; LV ESD = left ventricular end systolic volume; LA tr = transverse diameter of the left atrium; LA long = longitudinal diameter of the left atrium; LV EF = left ventricular ejection fraction; E = mitral early diastolic flow velocity; A = mitral A-wave velocity; E/A = ratio in mitral diastolic flow; A/a = ratio between the duration of mitral A-wave and duration of a-wave in pulmonary venous flow; s/d = ratio between systolic and diastolic pulmonary venous flow; S_m_ = mitral annulus systolic tissue velocity; E_m_ = early mitral annulus diastolic tissue velocity; E/E_m_ = ratio between mitral early diastolic flow velocity (E) and early mitral annulus diastolic tissue velocity (E_m_); Phase 0 = at baseline; Phase 1 = three months after the patients had received the second dosage of melphalan; Phase 2 = two years after successful double autologous HSCT; Phase 3 = six years after double autologous HSCT; Normal values^10^,^11^; PE = pericardial effusion; MR = mitral regurgitation
